# Music Therapy in the Neonatal Intensive Care Unit: A Center’s Experience with Program Development, Implementation, and Preliminary Outcomes

**DOI:** 10.3390/children11050533

**Published:** 2024-04-29

**Authors:** Carmina Erdei, Kim Schlesinger, Meredith R. Pizzi, Terrie E. Inder

**Affiliations:** 1 Department of Pediatrics, Division of Newborn Medicine, Brigham and Women’s Hospital, 75 Francis St, Boston, MA 02115, USA; kim@romanmusictherapy.com (K.S.); mpizzi@romanmusictherapy.com (M.R.P.); terrie.inder@choc.org (T.E.I.); 2 Pediatrics, Harvard Medical School, 25 Shattuck St, Boston, MA 02115, USA; 3 Department of Pediatrics, Division of Neonatology, Children’s Hospital of Orange County, 1201 W La Veta Ave, Orange, CA 92868, USA; 4 Pediatrics, University of California Irvine, 1001 Health Sciences Rd, Irvine, CA 92697, USA

**Keywords:** music therapy, NICU, music-based interventions, music medicine

## Abstract

Background: The role of music in the NICU continues to evolve, with recent studies documenting the positive impact of music therapy for hospitalized infants and families. With many potential benefits and no substantial adverse effects reported to date in medically stable infants, we aimed to create a clinical guideline to integrate this therapy into the NICU operations. Methods: we launched and implemented a pilot music therapy clinical program within a subunit of a level-III NICU, building upon available evidence. Results: In this report, we describe our experience with initial program development and early outcomes in terms of population served, frequency of music therapy, and therapeutic modalities employed to implement service delivery. Conclusion: we highlight the importance of establishing practices that are aligned with currently available data and recommendations, in order to facilitate delivery of a safe, evidence-based, meaningful therapeutic experience with monitoring of preliminary effects of the therapy on all those involved in the experience.

## 1. Introduction

**Figure 1:**
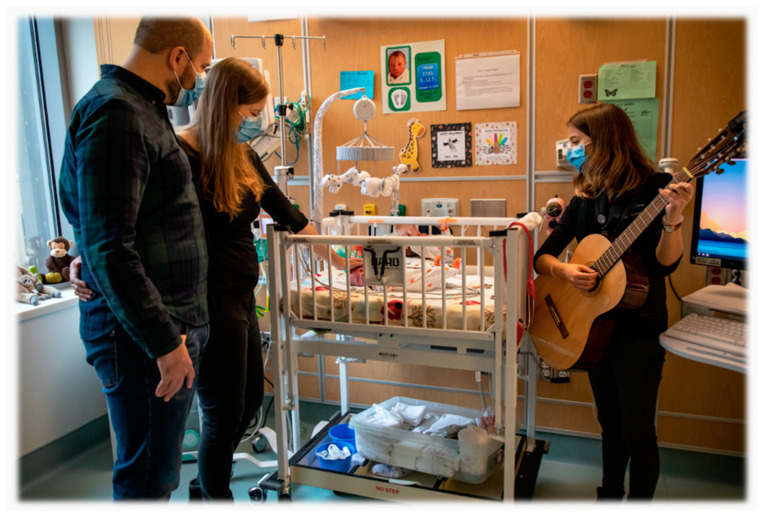
A MT-BC during a bedside music therapy session with an infant and family in the Growth and Development Unit.

A new father walks into the NICU to see his preterm infant, Patrick, after a full day at work. At the bedside, he greets his partner, Patrick’s mother, who has already spent hours that day learning about how to care for their son, and help him with feeding and other therapeutic strategies, with guidance from his interdisciplinary team. They’ve learned to ignore the sounds of a nearby alarm signaling the end of a nasogastric tube feed, a neighboring infant letting out a cry as his diaper is changed, a monitor’s beeps alerting a nurse to check on her patient. Patrick and his parents have taken part in several music therapy sessions during which the family has learned how to facilitate musical experiences matched to their infant’s developmental needs. They’ve also shared about their personal music preferences, their heritage, and the distance between themselves and the family who remains overseas in their home country. Today their music therapist enters to provide what has become a familiar and welcome musical space to hold Patrick, mom, and dad at the end of their day. Mom cradles Patrick, providing gentle rhythmic touch and rocking with subtle vestibular input, as dad takes a seat nearby. The music therapist begins to sing and play the family’s preferred Song of Kin [1]. While the parents dutifully go over the alternating vowel sounds, “too-rah loo-rah loo-rah”, with hopes to enhance their son’s auditory discrimination skills and perhaps even his later language development, the familiar sound of their chosen lullaby also brings peace, comfort, and connection with their baby and with each other. Evidence suggests that such meaningful moments are key for enhancing infant neurodevelopment and can also strengthen the parent–infant relationship, a construct so fragile and challenging to nurture within the walls of the NICU. Better yet, this experience creates space for a brief moment of reprieve for Patrick’s parents at the end of a long, busy day. The lullaby they sing together becomes a call out to their heritage, and a reminder of how far they’ve come building a life and a family far away from their own. (Figure 1). Very preterm (VP) infants, i.e., infants born below 32 weeks gestational age (GA) are often hospitalized for months in the Neonatal Intensive Care Unit (NICU) before becoming ready for discharge home with their family. For VP infants in the NICU, optimal brain development is contingent upon enriching sensory experiences occurring during their NICU hospitalization, which create the foundation for later cognitive, language, and social–emotional development [2,3,4,5]. From an auditory perspective, the NICU is an environment composed primarily of loud equipment sounds at a volume far exceeding recommended levels [6], silence, and overall reduced human interaction [4]. Concerns have been documented that limited desirable auditory and other parent-facilitated meaningful experiences, along with increased adverse auditory exposures (such as monitor alarms, mechanical ventilators and other equipment sounds, background noise during busy staff shifts, increased periods of silence), during a critical period can negatively impact brain maturation as well as neurodevelopment for hospitalized infants [4].

A proposed approach to addressing this auditory exposure gap in the NICU is by leveraging music therapy (MT), with the goal to offer enriching experiences for infants as well as their families. While conclusive evidence is still emerging [7,8], studies suggest that music therapy in the NICU may reduce stress in infants, as evidenced by enhanced autonomic stability during or after the intervention [9,10,11,12,13], and it may also improve feeding behaviors and state regulation [11]. Further, studies show that music therapy may enhance brain development and connectivity in networks underlying higher-order functions [14], promote acquisition of age-appropriate developmental milestones for hospitalized infants [15], and may have a beneficial effect in reducing parental anxiety [10] and family psychosocial distress [16]. Although more rigorous research is needed to generate stronger evidence, these preliminary data suggest that music therapy has the potential to enhance the experience of hospitalized infants and their families, and offers an opportunity to strengthen the parent–infant relationship [17] during a particularly vulnerable time in the NICU.

## 2. Program Development

In this report, we describe the conceptualization and rollout of a pilot music therapy program in a neurodevelopmental subunit embedded within a large level III NICU, specifically the Growth and Development Unit (GDU). The GDU program has been previously described [18], and it is designed to provide comprehensive, convalescent care to infants at high neurodevelopmental risk within a family-centered developmental care framework. The patients primarily cared for in the GDU include very preterm infants born before 32 weeks gestational age, and also infants with neurological or congenital conditions that require prolonged hospitalization and complex coordination of care. An interdisciplinary team provides a full spectrum of services and neurodevelopmental support for GDU infants and families. The unit consists of 18 beds in six newly renovated and redesigned semi-private bays, as well as two private rooms offered to families for rooming-in as available. As previously described [18], the GDU interdisciplinary team includes a core group of neonatologists with expertise in the neurodevelopmental care of medically complex infants, several full-time pediatric nurse practitioners, a team of neonatal nurses who care for their primary patients longitudinally during their NICU course, and a full spectrum of allied health disciplines, including physical, occupational, and feeding (speech-language pathology) therapy, nutrition, lactation, pharmacy, care coordination, social work, family support, and parent mental health therapy. This team works together with families to support each infant’s continued growth, development, and successful transition to the home of the family unit.

With a goal to integrate music therapy (MT) services within the GDU, the NICU departmental leadership sought collaboration with a local community music therapy agency (Roman Music Therapy Services, LLC, Wakefield, MA, USA). Initial goals focused on a programmatic assessment process to identify the needs, priorities, and potential barriers to implementation. Conceptually, the program was designed to focus on four goal areas: (1) promoting infant autonomic stabilization and developmental skill progression through contingent auditory experiences, (2) family education and coaching, (3) integrating the MT program in unit operations through collaboration with medical staff and allied health professionals, and (4) supporting research initiatives. The team focused on aligning this program’s goals and objectives with currently available data [7,8,19] and recommendations [20], in order to facilitate the delivery of a safe and evidence-based therapy within the NICU.

1. Promoting infant autonomic stabilization and developmental skills progression

The primary goal of the music therapy program is to nurture infant development. Through live music interventions presented by a board-certified music therapist (MT-BC) in collaboration with families, nursing staff, or allied health professionals, music therapy is implemented to support a variety of needs for the infants, including mitigating stress responses and increasing tolerance to environmental stimuli [21], promoting social interaction and early communication skills [22], developmental support [15,23], improving behavioral organization [11], improving sleep [24], supporting regulation and comfort [17], and supporting bonding and strengthening parent-infant relationships [25,26]. Specific interventions integrated in practice in our environment include Live Music Listening presented through humming, singing and/or steady guitar accompaniment, which family members are encouraged to engage in whenever able. These also include Developmental Music Interventions, which pair auditory stimuli with other sensory inputs such as high-contrast visual cards, hand motions, books, instruments, or gentle movement and tactile stimulation paired with lyrics. Music therapists are also trained in and deliver Multimodal Neurologic Enhancement [21,27] when appropriate, a 22-step progression used to systematically introduce gentle lullaby humming/singing, followed by infant stroking and then vestibular input. Of note, while current research has not reported significant adverse effects of MT for hospitalized infants, ongoing close monitoring of infant tolerance during MT sessions and documentation of any concerns or adverse effects remains a priority. Through these approaches, music therapists have the unique opportunity to support and coach parents to connect more intimately with their infants during these sessions, which in turn may empower parents to take the lead in other areas of care for their infants [26].

2. Family education and coaching

The music therapist can support the family-integrated developmental care model through several strategies, including working directly with parents at the bedside whenever feasible, and through occasional remote sessions when parents are unable to be present in the NICU. By creating space for, and facilitating a shared experience of, MT, music therapists have the opportunity to coach parents regarding the developmental needs of their infants, and even facilitate early parent–infant communication exchanges. Furthermore, they can offer parent education around reading and responding contingently to their infant’s cues through teachable moments that occur during music therapy sessions. Overall, music therapists have the valuable opportunity to coach families in honing the development of their infants’ sensory systems through early meaningful experiences, which represent essential building blocks for later neurodevelopment.

3. Collaborating with medical staff and allied health professionals

The MT program was conceptualized and rolled out with the goal to complement and enhance the established medical and neurodevelopmental services in the GDU. The music therapy team has subsequently partnered with the medical staff to create a clinical practice guideline in keeping with other unit policies and procedures. This guideline was developed in collaboration with interdisciplinary staff and describes a structured protocol for MT eligibility and delivery of service for patients and families in the NICU, with focus on the GDU population. Allied health collaborators, specifically the dedicated neonatal developmental therapy team in this unit, were identified as the optimal liaison to guide MT referrals.

4. Supporting research initiatives

In addition to direct clinical service, another aim of the program has been to partner with and support ongoing research initiatives in the NICU. This collaboration includes critical review of existing MT evidence, connecting with MT researchers at other institutions, and providing consultation into the design and implementation of research protocols involving music-based interventions and MT in the NICU.

In the early stages of this program, MT services were offered only to infants and families in the GDU. As the program grew and coverage expanded, MT was also considered for other select infants and families in the larger unit using a staged approach. Ongoing collaboration between the MT team, the medical team, and allied health professionals is key to refining the optimal referral criteria for referrals for patients within the larger NICU who can benefit from expanded MT services.

## 3. Program Implementation and Operational Considerations

1. Staffing, training, and onboarding

Adequate staffing with trained, board-certified MTs is essential to a successful program model. A board-certified music therapist (MT-BC) is a professional with a music therapy degree from an approved university program [28], who subsequently underwent a clinical internship [29], and has passed a national certification exam [30]. While this training equips a music therapist to take on many roles within the MT field, additional training is encouraged for specialized settings such as the NICU. To prepare for the delivery of services in the NICU, the music therapists in this unit pursued additional continuing education opportunities such as through the National Institute for Infant and Child Medical Music Therapy [31] and The Louis Armstrong Department of Music Therapy at Mount Sinai Beth Israel in New York, NY [32]. This provided a depth of understanding across the team in regard to interventions and considerations particular to the NICU environment, as well as across different training programs and approaches within the field of MT.

The therapists engaged in our NICU subsequently underwent an onboarding phase, during which there were ample opportunities to meet and work with other disciplines, including medical, nursing, physical therapy, occupational therapy, feeding therapy/ speech and language pathology, and the social work/care continuum. Gaining a working knowledge of the role and scope of work of each of these subspecialties within the NICU facilitated close interdisciplinary partnerships and allowed the music therapists to integrate the goals and rollout of the MT program within the existing operations of the unit.

2. Building and enhancing partnerships

The primary goal of our partnership was to improve health outcomes through a synergetic relationship that creates outcomes that are greater than individuals or organizations could accomplish working independently [33,34]. Key aspects need to be considered to enhance such synergetic partnerships [34], such as resources, partner characteristics, relationships among partners, partnership characteristics, and the external environment. All these determinants influenced the initial success of our program. Clarity around expectations was essential between the hospital and agency partners as we developed plans for onboarding and training, service delivery, and medical record documentation, to build a successful program. Through ongoing discussion with the medical team, hospital administrators, other clinical providers on the unit, music therapists, and MT agency supervisors and administrators, we established reasonable, mutually agreed-upon targets for service delivery. Flexibility in contractual language provided time for consultation and program development. This was essential as the MT team developed documentation forms and educational materials for staff and families, and worked through other implementation logistics. Regularly scheduled meetings with the teams from both organizations created a positive atmosphere of collaboration and provided opportunities to make adjustments as needed [35]. These consistent touchpoints allowed the teams to celebrate successes and address any challenges promptly.

3. Operational Considerations

During the first year this program was rolled out (Phase One), the teams focused on the following objectives: (1) creating and completing an internal training and onboarding plan; (2) developing a patient referral/triage process; (3) determining the workflow for the music therapy team from referral to service delivery to documentation strategies in the hospital medical record; (4) defining music therapy interventions drawing on the best available evidence, and outlining them in a clinical practice guideline; (5) collaborating with and educating medical and allied health professionals regarding the role of MT in the NICU environment; and (6) creating resources and other MT materials for parents. A medical director was also identified to oversee the rollout and implementation of the program, integrate the MT program into the NICU operations, align the program with the culture of the unit, assist through any challenges, and serve as an advocate, sponsor, partner, and key liaison with the MT team. Upon entering the second year of NICU MT program operation (Phase Two), the focus shifted towards increasing service delivery, efficiency, streamlining referrals, and optimizing the ordering and documentation processes in the medical record. From a financial perspective, this MT program has been made possible in our unit through philanthropic support. Presently, this program functions as a non-billable, enriching intervention available for eligible infants and families in the NICU following an established clinical practice guideline.

With regard to hours of service delivery, the program was rolled out with coverage corresponding to 12 h of weekly service delivery in Phase One. Operationally, this translated to 1.5 days of coverage per week (one 8-h and one 4-h shift), with one music therapist serving the GDU subunit, which encompasses up to 20 beds (average census 16–18 patients). In later phases, a second therapist was added to the team to provide increased services inclusive of weekend hours whenever feasible, to facilitate enhanced accessibility and support for families.

## 4. Initial Program Metrics and Outcomes

1. Program rollout and early outcomes

Within the 6-month period following the program’s implementation (September 2021–March 2022, Period 1), 76 preterm infants received at least one session of MT service in the GDU (Table 1). This number increased to 98 infants serviced during the second 6-month period (Period 2: February 2022–August 2022), and 100 infants during months 13–18 of program implementation (Period 3: September 2022–March 2023). The total number of infant music therapy sessions that occurred during Periods 1, 2, and 3 (including infants who received multiple sessions) were 241, 265, and 281 sessions, respectively, reflecting a gradual increase in service hours and capacity as the program became more established. On average, each infant received approximately 3 music therapy sessions during their stay, with the individual infant sessions ranging between 1 and 11 sessions. In keeping with the unit clinical practice guideline, infants were eligible to engage in MT service after 32 weeks postmenstrual age, with a range of age at first session being between 32 and 45 weeks of GA in our cohort.

The specific music therapy interventions utilized with infants in close collaboration their caregivers included the following: live music for soothing and relaxation (MT-BC-facilitated infant directed singing, live music listening), parental live singing with MT-BC coaching [25], song of kin [1], live music for developmental stimulation (musical book, auditory localization, musical visual stimulation through hand motions or visual aids), procedural support [36], responsive musical stimulation, musical tactile stimulation (holding/gentle rhythmic touch), musical and vestibular stimulation, and multimodal neurologic enhancement [21,27,37,38]. The most consistent approach across all music therapy sessions was contingent live music listening of some form, whether presented through humming and vocal singing or with the additional component of guitar accompaniment per infants’ tolerance and following their behavioral cues. Additional opportunities for guided parental engagement were incorporated and encouraged within each session as appropriate. Of note, a major priority was to involve families in most of the music therapy sessions as able (66–73% of sessions, Table 1), in order to tailor the content of each session to reflect and align with each family’s goals and diverse needs whenever possible.

2. Family and staff informal feedback

Parent engagement opportunities ranged from holding their infant and engaging in discussion or coaching with the music therapist, to active music-making through parents singing or humming. Across the first 18 months of the music therapy program, 70% of sessions included the presence of at least one family member or caregiver. Family engagement was observed to advance as parents gradually became more comfortable with the music therapist and the techniques used, with some parents taking the opportunity to independently present lullabies that were unique and meaningful to their family or translate into their native language. One family shared, “We were so excited when we remembered it was Friday ‘music day’. We always enjoy and look forward to your visits”. Another family shared the joy that music brought into their lives in the hospital, reflecting: “This was the best day!”. As we informally observed many parents reflecting on how music therapy helped them enjoy a cherished moment of relaxation and connection with their infant in the midst of an otherwise difficult time, we aimed to continue to support parents in strengthening their relationship with their infants through MT [17], establish meaningful routines, and hone skills that will remain valuable beyond discharge from the NICU [39].

Medical, nursing, and allied health collaborators who had the opportunity to observe and/or take part in music therapy sessions have also expressed their support and gratitude for the implementation of MT services into the unit. A nurse on the unit articulated, “Music is so important; singing to babies is timeless, people have always been singing to their babies”. Other nurses reflected on the benefits music has provided during their workdays. One nurse shared “It reminds you of the beauty and humanity with everything that’s going on. It’s so pure and it helps everyone”; another stated, “Now the whole room is calmer and more peaceful”.

## 5. Strengths, Challenges, Limitations

Since its inception and initial rollout in September 2021, we continue to progress towards our goal to integrate a new neonatal service—the MT program—within the NICU model of care. We have achieved this goal through creating and training a specialized team, establishing an evidence-based, standardized practice guideline outlining the program role, scope, and clinical operations, and building relationships with interdisciplinary staff and families, all of which were foundational to integrating this program into the fabric and culture of our NICU.

Throughout this process, several challenges have risen which warrant further discussion. A key consideration consists of ensuring access to sustainable financial support for a new MT program in the NICU. In our environment, this program has been supported through philanthropy, with close collaboration of clinical and administrative leadership with the institutional Development office to ensure the program’s financial feasibility and sustainability. Furthermore, while MT services may be of benefit to a wide range of patients beyond the GDU subunit, we note limitations in terms of the MT team’s capacity to service a defined number of patients within the contracted service hours. Ongoing work is dedicated to incrementally expanding the eligibility criteria for MT services beyond the GDU population, in order to offer this service to other patients and families for whom music therapy could provide benefit within the available service hours. Examples of eligibility criteria expansion in keeping with current evidence include incorporating MT in palliative care plans [40,41], or for post-procedural support as able [36]. Further, while studies that evaluated MT use in the NICU did not report adverse events [7], we note that most studies have been conducted on medically stable infants following rigorous research protocols. Very limited data is currently available regarding the safety and feasibility of clinical MT services for higher-risk populations, including infants with advanced neurological injury [42] or other medical acuity and complexity. This is an area that deserves further investigation, as stronger evidence is needed regarding the safety, feasibility, and efficacy of music therapy in these vulnerable populations to better inform clinical practice. Lastly, while this report descriptively presents our experience with initial program development and early outcomes in terms of population served and therapeutic modalities employed, we recognize that an objective, quantitative assessment of program outcomes, including clinical outcomes and formal documentation of parent and staff experiences, are important next steps. We also acknowledge that there are other strategies beyond what we have described here, such as leveraging the use of devices [43] or recorded music interventions [44] that may facilitate safe and effective delivery of music experiences for infants hospitalized in the NICU, which can also be considered in further work.

## 6. Conclusions

The field of MT and its role in the NICU continues to evolve, with recent literature documenting the preliminary positive impact of this therapeutic modality on hospitalized infants and their families. With many potential benefits and no known adverse effects reported to date in MT protocols for eligible, medically stable infants, there is a solid argument in favor of bringing this therapy into the NICU. Yet, unanswered questions and knowledge gaps remain, which call for rigorous clinical trials to further study the effects of standardized MT on clinically relevant outcomes of hospitalized babies and their families, in order to create a stronger base of evidence supporting this service. This will be critical for further establishing this approach as not just a “nice to have” in the NICU, but rather, an evidence-based therapy that all eligible hospitalized infants should have equitable access to. We, along with other units across the country, have proceeded to launch a MT clinical program based on available knowledge, to bring this therapy intervention to hospitalized infants and families while awaiting stronger evidence to further inform optimal service delivery. We highlight the importance of establishing practices and protocols that are aligned as best as possible with currently available data and recommendations to facilitate delivery of a safe, evidence-based therapy, along with monitoring of the impact on all those involved in the experience.

## Figures and Tables

**Table 1:** Clinical music therapy program outcomes at 6, 12, and 18 months after rollout (September 2021–March 2023).

	Period 1 1–6 Mo	Period 2 7–12 Mo	Period 3 13–18 Mo	Total 18 Mo
# infants receiving MT	76 infants	98 infants	100 infants	274 infants
Average sessions/infant	3.3	2.7	2.8	2.9
Total # of sessions	241	265	281	787
# of sessions/patient	1–11	1–6	1–10	1–11
Mean GA for first MT	36w5d	36w1d	36w3d	36w3d
Mean Birth GA	31w1d	31w5d	32w1d	31w5d
Parent engagement in MT	71%	66%	73%	70%

Abbreviations: Mo: months; w: weeks; d: days.

## Abbreviations

GA	Gestational Age
GDU	Growth and Development Unit
MT	Music Therapy
MT-BC	Music Therapist-Board Certified
NICU	Neonatal Intensive Care Unit
PMA	Post-Menstrual Age
VP	Very Preterm
